# Impact of the COVID-19 pandemic on glaucoma surgery in German hospitals

**DOI:** 10.1007/s00417-025-06787-9

**Published:** 2025-03-07

**Authors:** Philip Keye, Charlotte Evers, Timothy Gläser, Philip Braun, Patrick Thelen, Daniel Böhringer, Stefan Johann Lang, Thomas Reinhard, Jan Lübke

**Affiliations:** 1 Eye Center, Medical Center – University of Freiburg, Faculty of Medicine, University of Freiburg, Freiburg, Germany; 2 Department of Ophthalmology, St. Franziskus-Hospital, Münster, Germany; 3 Department of Ophthalmology, Brandenburg Medical School, University Hospital Brandenburg, Brandenburg an der Havel, Germany; 4 Klinik Für Augenheilkunde, Universitätsklinikum Freiburg, Killianstraße 5, 79106 Freiburg, Germany

**Keywords:** Glaucoma, Surgery, Germany, Hospitals, COVID-19, Pandemic

## Abstract

**Purpose:**

To assess the quantitative changes in surgical glaucoma care in German hospitals between 2019 and 2022 with special focus on the impact of the COVID-19 pandemic on overall volume and trends within glaucoma surgery.

**Methods:**

The quality reports of The Federal Joint Committee (G-BA), containing information on the quantity of surgical glaucoma procedures of 296 German hospitals were obtained in machine-readable form for the years 2019, 2020, 2021 and 2022. We analyzed the annual numbers and proportions of different glaucoma surgery types as categorized by German OPS codes, with special focus on 2020, the first year of the COVID-19 pandemic in Germany.

**Results:**

The total number of surgical glaucoma procedures in German hospitals in 2020 decreased by 8.5% compared to 2019 and recovered to pre-pandemic levels in 2021. Within filtration surgery, the number of classic trabeculectomy steadily declined while bleb-forming filtration devices were used more frequently. In all four years, cyclodestructive procedures were the most frequently performed interventions overall.

**Conclusion:**

The impact of the COVID-19 pandemic on overall surgical volume, especially in 2020, was substantial but overall moderate and transient. The trend towards minimally invasive procedures and bleb-forming filtration devices accelerated after 2020, resulting in a pronounced decline of classic filtration surgery, such as trabeculectomy.

**Supplementary Information:**

The online version contains supplementary material available at 10.1007/s00417-025-06787-9.

## Abstract (highlights)

**Key messages:**

*****What is known***:**

- In 2020, safety precautions regarding the emergence of the SARS-CoV-2 virus led to cancellations and postponements of elective surgical procedures in Germany, including glaucoma surgery.

*****What is new***:**

- The total volume of glaucoma surgery procedures in 2020 declined by approximately 8.5% compared to 2019 and recovered to pre-pandemic levels in 2021.

- A pre-existing trend towards implant-based filtration surgery was consolidated.

**Supplementary Information:**

The online version contains supplementary material available at 10.1007/s00417-025-06787-9.

## Introduction

Glaucoma is a worldwide leading cause of blindness. With aging populations, its prevalence is expected to further increase in the future [1]. The disease is characterized by a progressive loss of retinal ganglion cells, a characteristic cupping of the optical nerve head and ultimately visual field loss [2]. Risk factors include older age, family history of glaucoma and elevated intraocular pressure. To date, lowering the intraocular pressure remains the only evidence-based therapy [2, 3]. Medical management incorporates substances, that either decrease outflow resistance or the production of aqueous humor. Alternatively, laser procedures or incisional surgery may be performed. Laser procedures include selective laser trabeculoplasty (SLT) or cyclodestructive approaches such as cyclophotocoagulation. Regarding surgical procedures, classic filtration surgery (trabeculectomy) is still widely considered to be the standard of care [4]. However, stent-based filtration procedures such as the XEN® gel stent (Allergan, an Abbvie company, Irvine, CA, USA) or the PreserFlo® MicroShunt (Santen Pharmaceutical Co. Ltd.), that follow the same principle of creating a shunt between the anterior chamber and the subconjunctival space, have been shown to increase in number over the last decade [5]. Non-penetrating procedures (e.g. deep sclerectomy) avoid a direct shunt between the anterior chamber and the subconjunctival space and bare less risk of postoperative hypotony compared to filtration surgery. All procedures with subconjunctival drainage are at risk of failure due to fibrosis of the conjunctival bleb. Needling or open revision surgery may be performed to reestablish flow through the shunt. Microinvasive glaucoma surgery (MIGS) is an umbrella term for a number of procedures that share certain characteristics: MIGS procedures are in general relatively easy and safe to perform and their ab interno approach requires only small corneal incisions without the need for conjunctival dissection. For example, enhancing trabecular outflow can be achieved with anterior chamber angle (ACA) stents such as the iStent® (Glaukos Corporation, Laguna Hills, CA, USA) and the Hydrus® Microstent (Alcon, Fort Worth, TX, USA) that are implanted into Schlemm’s canal [6]. A trabeculotomy may be performed using the Kahook Dual Blade® (New World Medical, Rancho Cucamonga, CA, USA) or Trabectome® (MicroSurgical Technology, Redmond, WA, USA) among other options [4]. Another MIGS procedure, the MINIject (iSTAR Medical SA, Wavre, Belgium) device opens the suprachoroidal space for outflow of aqueous humor. Glaucoma drainage devices such as the Ahmed glaucoma valve (New World Medical, Rancho Cucamonga, CA, USA), the Baerveldt glaucoma implant (Abbott Medical Optics, Santa Ana, CA, USA) or Paul glaucoma implant (Advanced Opthalmic Innovations, Singapore, Republic of Singapore) require a larger conjunctival dissection compared to trabeculectomy as they are placed rather posteriorly, partly tucked under the recti muscles.

Due to newly introduced procedures, glaucoma surgery as a whole underwent significant change over the last years. As the number of trabeculectomies consistently declined, glaucoma tube surgery and especially MIGS procedures are performed more frequently [5, 7, 8].

In early 2020 the global spread of SARS-CoV-2 and the resulting COVID-19 pandemic forced hospitals worldwide to reserve capacities for potential COVID patients. Consequently, elective patient contacts and many elective surgical procedures were postponed or cancelled [9]. Regarding ophthalmology, a notable decline in patient visits and surgical volume has been reported for cataract surgery [10], refractive surgery [11] and glaucoma surgery [12]. While delayed diagnosis and treatment may not necessarily be harmful, for example in cataract cases, patients with chronic progressive diseases like glaucoma may suffer from preventable vision loss [13]. Consequently, eye care providers adapted and concepts like telemedicine consultations were developed in order to safely perpetuate eye care [14]. In addition to the quantitative effect of the COVID-19 pandemic, glaucoma surgery has also been shown to have changed qualitatively. Data from Italy and the United Kingdom showed a decline in trabeculectomies and a trend towards MIGS procedures, that may require less frequent patient visits during the postoperative phase [15, 16].

In this study, we report on changes of glaucoma surgery volume and trends within surgical glaucoma care during and after the COVID-19 pandemic, analyzing data from over 400 German hospitals.

## Methods

All data were obtained from the annual hospital quality reports of The Federal Joint Committee (G-BA). The quality reports from 2019 to 2022 were requested in machine-readable form and relevant data were converted into a relational database (https://www.g-ba.de/themen/qualitaetssicherung/datenerhebung-zur-qualitaetssicherung/datenerhebung-qualitaetsbericht/servicedateien/). The hospital quality reports were used only partially or in extracts. For a complete, unchanged presentation, see https://www.g-ba.de/. The quality reports include pooled data of 434 German hospitals with ophthalmology departments. Out of these, 296 institutions reported a glaucoma surgery OPS code at least once (see supplementary information for the full list of glaucoma procedure OPS codes). The OPS system is the German version of a classification of medical procedures. It is maintained and published by the Federal Institute for Drugs and Medical Devices (BfArM, https://www.bfarm.de/DE/Home/_node.html). For this analysis, we selected OPS codes that were considered most relevant for surgical glaucoma management in Germany (Table 1). The analysis focused on year-to-year changes in absolute numbers and proportions within the defined groups. We specifically analyzed the following surgical procedures and their corresponding OPS codes: Filtration surgery, including trabeculectomy (e.g. 5–131.01) and implant-based variants (5–131.63). Bleb revision (5–131.40). MIGS procedures, including anterior chamber angle devices (5–131.61), suprachoroidal devices (5–131.62) and variants of trabeculotomy (e.g. 5–133.80 and 5–133.81). Non-penetrating procedures, including deep sclerectomy (e.g. 5–134.1) and viscocanaloplasty (5–134.2). Glaucoma drainage implants (5–131.64). Cyclodestructive procedures, including cyclocryocoagulation (5–132.1) and variants of cyclophotocoagulation (e.g. 5–132.2). Minor outpatient treatment procedures (e.g. SLT) were not included since the quality reports do not contain data of private practices.

**Table 1:** Detailed list of all included OPS codes and the corresponding procedures

OPS	Procedure
5–131.0	Goniotrepanation, Trabeculectomy
* 5–131.00*	* … without antifibrotic agents*
* 5–131.01*	* … with antifibrotic agents*
* 5–131.0x*	* Others*
5–131.40	Bleb revision
5–131.61	Sutureless implant, drainage into the ACA
5–131.62	Sutureless implant, drainage into the suprachoroidal space
5–131.63	Sutureless implant, drainige into the subconjunctival space
5–131.64	Suture fixated implant, drainige into the subconjunctival space
5–132.1	Cyclocryocoagulation
5–132.2	Cyclophotocoagulation
* 5–132.20*	* Open*
* 5–132.21*	* Endoscopic*
* 5–132.22*	* Transscleral*
* 5–132.2x*	* Others*
5–133.80	Trabeculotomy ab interno (Laser)
5–133.81	Trabeculotomy ab interno (Electroablation)
5–133.8x	Trabculotomy ab interno (others)
5–134.1	Deep sclerectomy
* 5–134.10*	* … without antifibrotic agents*
* 5–134.11*	* … with antifibrotic agents*
5–134.2	Viscocanaloplasty

Between 2019 and 2024 the relevant OPS codes remained unchanged. Notably, OPS code 5–131.64 (“Suture fixated implant, drainage into the subconjunctival space”) was newly introduced in 2019.

In some cases, different procedures fall under one OPS code and can therefore not be further distinguished. For example, OPS 5–131.61 (“Sutureless implant, drainage into the anterior chamber angle”) includes the iStent and the Hydrus microstent and OPS 5–131.63 (“Sutureless implant, drainage into the subconjunctival space”) includes XEN and PreserFlo devices. In other cases, different OPS codes were grouped. For example, OPS codes 5–131.1, 5–131.20, 5–131.21, 5–131.22 and 5–131.2 × were summed up under cyclodestructive procedures. For all defined groups, see Table 2.

**Table 2:** Defined groups of procedures (left) and included procedures and OPS codes for each group (right)

Group		Procedures (OPS codes)
Filtration surgery	*Classic*	Goniotrepanation, Trabeculectomy (5–131.00, 5.131.01, 5–131.0x)
	*Implant-based*	PreserFlo/ XEN (5–131.63)
Bleb revision		Bleb revision (5–131.40)
MIGS	*ACA implants*	iStent/ Hydrus (5–131.61)
	*Suprachoroidal implants*	MINIject (5–131.62)
	*Trabeculotomy*	Excimer Laser Trabeculotomy (5–133.80) Trabectome (5–133.81) Other ab interno trabeculotomies (5–133.8x)
Non-penetrating procedures		Deep sclerectomy (5–134.1, 5–134.10, 5–134.11) Viscocanaloplasty (5–134.2)
Glaucoma Drainage Implants		Ahmed/ Baerveldt/ Paul GDI (5–131.64)
Cyclodestructive procedures		Cyclocryocoagulation (5–132.1) Cyclophotocoagulation (5–132.2, 5–132.20, 5–132.21, 5–132.22, 5–132.2x)

There is no consistent definition of MIGS procedures. For this work, XEN and PreserFlo devices were excluded from the MIGS group and analyzed separately as a subgroup within filtration surgery.

Concerning revision surgery after filtering surgery, only OPS code 5–131.40 (“Bleb revision”) was included in the analysis. This code includes bleb needling as well as open bleb revision surgery. Both procedures may be performed following classic filtration surgery, as well as after filtration surgery with implants. OPS code 5–131.42 (“suture lysis”) was not included in the analysis as we consider it to be of minor interest regarding the scope of this work. All graphs were created using the R system (A Language and environment for statistical computing. R Foundation for Statistical Computing, Vienna, Austria).

## Results

**Fig. 1:**
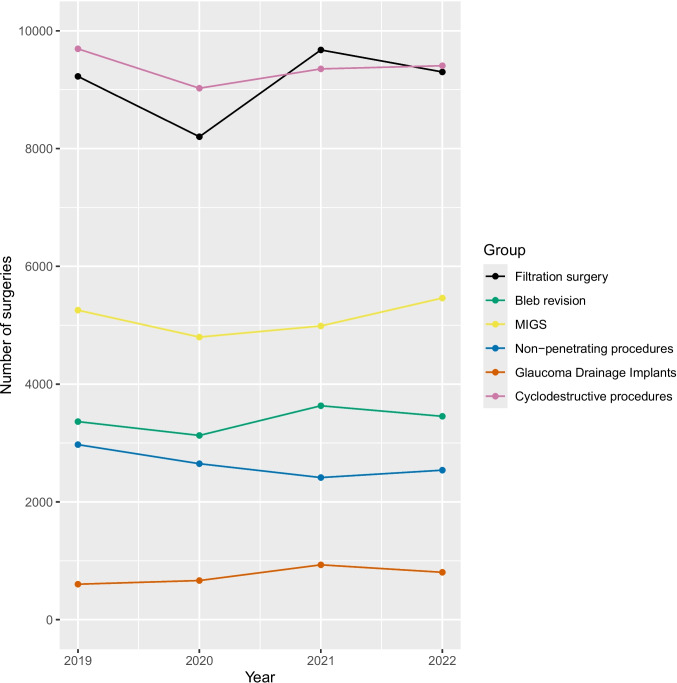
Changes in the total number of procedures per group over the observation period

The total number of procedures in 2019 was 31,116 and declined to 28,468 in 2020 (−8.5%). In 2021, the number increased again to nearly 31,000 and stagnated at that level in 2022 (Table 3). All subgroups except for glaucoma drainage devices declined in 2020 (Fig. 1).

**Table 3:** Total number of procedures, number of procedures per group, and proportion of each group relative to the total for each year

	2019	2020	2021	2022
Total	31,116 (100%)	28,468 (100%)	30,995 (100%)	30,968 (100%)
Filtration surgery	**9,226 (29.7%)**	**8,201 (28.8%)**	**9,675 (31.2%)**	**9,301 (30.0%)**
* Classic*	*5,818 (18.7%)*	*5,161 (18.1%)*	*5,129 (16.5%)*	*4,134 (13.3%)*
* Implant-based*	*3,408 (11.0%)*	*3,040 (10.7%)*	*4,546 (14.7%)*	*5,167 (16.7%)*
Bleb revision	**3,364 (10.8%)**	**3,129 (11.0%)**	**3,633 (11.7%)**	**3,454 (11.2%)**
MIGS	**5,256 (16.9%)**	**4,799 (16.9%)**	**4,988 (16.1%)**	**5,462 (17.6%)**
* Anterior chamber angle implants*	*3,566 (11.5%)*	*3,113 (10.9%)*	*3,252 (10.5%)*	*3,482 (11.2%)*
* Suprachoroidal implants*	*90 (0.3%)*	*32 (0.1%)*	*95 (0.3%)*	*385 (1.2%)*
* Trabeculotomy*	*1,600 (5.1%)*	*1,654 (5.8%)*	*1,641 (5.3%)*	*1,595 (5.2%)*
Non-penetrating procedures	**2,973 (9.6%)**	**2,649 (9.3%)**	**2,414 (7.8%)**	**2,539 (8.2%)**
Glaucoma Drainage Implants	**603 (1.9%)**	**665 (2.3%)**	**932 (3.0%)**	**805 (2.6%)**
Cyclodestructive procedures	**9,694 (31.2%)**	**9,025 (31.7%)**	**9,353 (30.2%)**	**9,407 (30.4%)**

### Filtration surgery and bleb revision surgery

**Fig. 2:**
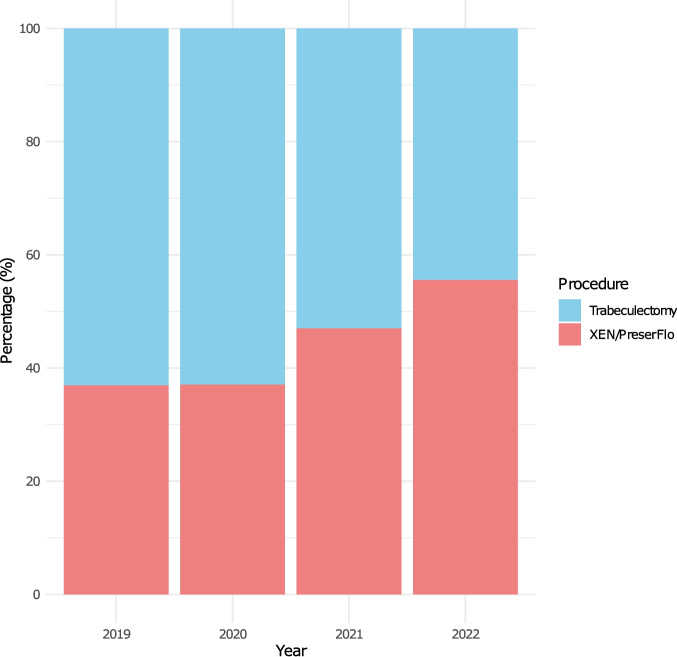
Changes of the distribution between classic (blue) and implant-based (red) filtering procedures over the observation period

The total number of filtration surgeries dropped from 9226 procedures in 2019 (29.7% of total) to 8201 in 2020 (28.8% of total), equalling a decline of 11.1%. In 2021, 9675 filtering procedures were performed, an increase of 18% compared to the previous year. With 9301 procedures in 2022, the number reached pre-pandemic levels again. Within the filtration surgery group, trabeculectomy numbers consistently declined between 2019 and 2022 (Fig. 2). While 63.1% of all filtration procedures in 2019 were trabeculectomies, the fraction was 44.4% in 2022. Filtration devices strongly increased in number after the initial dip in 2020 with an overall rise of 51.6% between 2019 and 2022.

The number of bleb revision procedures showed a moderate decrease of approximately 7% in 2020 and exceeded pre-pandemic levels in 2021 (up 8%) and 2022 (up 2.7%).

### MIGS procedures

Between 2019 and 2022, MIGS accounted for 16.1% (2021) to 17.6% (2022) of all procedures. Compared to 2019, MIGS procedures initially dropped by 8.7% in 2020 and recovered to approximately pre-pandemic levels during the following two years. The distribution between trabeculotomies, ACA stents and suprachoroidal implants remained roughly stable except a significant increase of suprachoroidal implants in 2022 (up over 400%) compared to the previous years.

### Non-penetrating procedures

With 2973 procedures in 2019, non-penetrating glaucoma surgery accounted for 9.6% of total procedures in that year and the portion varied between 7.8% (2021) and 9.3% (2020) over the following years. In 2020, the total number of non-penetrating procedures declined by 10.9% and never exceeded pre-pandemic levels during the observation period.

### Glaucoma drainage implants

The number of glaucoma drainage implants did not decline in 2020. Instead, from 603 procedures in 2019, it increased to 665 in 2020 (up 10.3%) and 932 in 2021 (up 54.6%). In 2022, the number dropped to 805 procedures, still exceeding pre-pandemic levels by 33.5%.

Glaucoma drainage implants accounted for 1.9% to 3.1% of all procedures during the observation period.

### Cyclodestructive procedures

Cyclodestructive procedures were the most frequently performed surgery in 2019 (9694 procedures, 31.2% of total), 2020 (9025 procedures, 31.7% of total) and 2022 (9407 procedures, 30.4% of total). In 2020, the number of cyclodestructive procedures dropped by approximately 7% compared to 2019.

## Discussion

In this work, the total volumes of surgical glaucoma procedures and trends within surgical glaucoma care between 2019 and 2022 were analyzed using annual hospital quality reports that contain pooled data of 296 German institutions that performed surgical glaucoma procedures. Due to regulatory requirements and specifications regarding reimbursement, surgical glaucoma care in Germany is predominantly provided by hospitals. Minor glaucoma laser procedures like LPI and SLT were excluded from the analysis since a significant portion of these procedures is performed in private practices. With this restriction, the data presented in this work largely represent surgical glaucoma care between 2019 and 2022 in Germany as a whole.

The aim of this study was to analyze changes in glaucoma surgery volume between 2019 and 2022, as the COVID-19 pandemic reached Germany in 2020 and hospitals had to withhold beds, material and personnel for critically ill patients. Published data from German hospitals showed that total case numbers decreased by 13% in 2020 with a maximal decline of minus 30% between March 9th and May 24th 2020 [17]. Similarly, elective surgical procedures were cancelled or postponed worldwide [18], including eye surgery. For example, cataract surgery in the UK was halted between March and June 2020 [19] and refractive surgery volume decreased significantly in the USA and elsewhere [11]. A single-center analysis from an Italian hospital by Longo et al. showed a decline of glaucoma surgical procedures by approximately 30% [12]. For Germany, our data suggest an overall glaucoma surgery volume decline in 2020 by 8,5% compared to 2019. While our data do not allow us to break the numbers down to surgeries per month, it is reasonable to assume that most cancellations happened during the first COVID-19 lockdown between March and May 2020. The relatively minor decline for the whole year may be influenced by multiple factors: glaucoma surgeries usually are not strictly elective procedures as prolonged episodes of uncontrolled intraocular pressure may cause irreversible damage to the optical nerve head. Accordingly, a crisis strategy paper, authored by heads of ophthalmology departments in Germany and the German Society of Ophthalmology (DOG) classified the progressive and medically uncontrollable glaucoma as a relative surgical emergency [20]. Glaucoma surgery may therefore have been prioritized over other procedures, e.g. cataract surgery. Additionally, the German healthcare system never experienced an overload of COVID-19 patients that would have required resources from ophthalmology departments to be allocated elsewhere, allowing hospitals to proceed with eye surgery under appropriate safety precautions relatively quickly.

Besides the changes in overall surgical volume, we analyzed how the numbers of specific procedures and groups of similar procedures changed over the observation period. For Germany, it has been shown that the number of classic filtration surgeries (e.g. trabeculectomy) consistently decreased over the last years [5]. Our data suggest that the COVID-19 pandemic aggravated this trend with a decline of classic filtration surgery by approximately 30% between 2019 and 2022. On the other hand, a strong shift within filtration surgery towards stent-based procedures such as the XEN gel stent and the PreserFlo Microshunt is apparent. This may be due to the supposedly more favorable safety profile of these procedures compared to classic trabeculectomy and surgeons may have moved towards these alternatives to reduce the number of postoperative patient visits. Notably, the relatively stable numbers of bleb revisions suggest a similar failure rate of classic and modern filtration surgeries. Another notable trend is the increase of glaucoma drainage implants, especially in 2021 and 2022. As these devices tend to be used in the management of advanced glaucoma, this may indicate that patients presented with more severe glaucoma in 2021 and 2022 due to reduced access to eye care during COVID. The observation that glaucoma patients are especially prone to suffer preventable loss of vision due to delayed ophthalmic care may support this theory [13]. Within the MIGS group, a notable change occurred in 2022, when the MiniJect implant was approved in Germany, reflecting the demand for innovative surgical technologies.

While the decline in overall surgical volume in 2020 can be attributed with relatively little doubt to the impact of the COVID-19 pandemic, the reasons for specific trends within glaucoma surgery are more complex. For Germany, the volume of classic filtration surgery (e.g. trabeculectomy) has been reported to decrease since 2008, while the numbers of MIGS and implant-based procedures in general are steadily increasing [5]. The willingness of surgeons to rapidly adopt new technologies suggests a high demand for alternatives to classic surgical procedures, independent form disruptors like the COVID-19 pandemic. However, we hypothesize that this development may have been accelerated during the pandemic as surgeons and patients may have favored procedures with less frequent postoperative visits.

In summary, the impact of the COVID-19 pandemic on overall surgical volume in German hospitals was moderate, with an 8.5% decline in overall glaucoma surgery volume compared to the previous year, and transient, with a recovery to pre-pandemic levels in the following years 2021 and 2022. Within glaucoma surgery, trends towards modern filtration procedures were consolidated while classic procedures like trabeculectomy further decreased in number. The increase of glaucoma drainage implants in 2021 and 2022 may imply an increase of patients presenting with advanced glaucoma due to delayed ophthalmic care during the pandemic. It is important to emphasize that these findings are based on data from the German healthcare system and may not be applicable to other countries or regions. However, considering this study’s results and reflecting on glaucoma care during the first year of the COVID-19 pandemic, some lessons can be drawn for glaucoma care providers in general. Firstly, the pandemic highlighted the importance of adaptable healthcare systems that can quickly adjust to unforeseen circumstances. Secondly, clear guidelines and protocols are needed to prioritize surgical procedures during times of crisis, ensuring that patients with a high risk of irreversible damage receive timely treatment. Finally, the consolidation of trends towards less invasive procedures, potentially requiring less frequent postoperative visits, might represent a strategy to mitigate the impact of future disruptions. Future research could focus on exploring the long-term outcomes of these adapted approaches.

### Limitations of the study

Since the numbers in the quality reports are based on self-reported OPS codes, incorrect encoding of surgical procedures may have resulted in inaccurate data. However, correct coding is in the hospitals' best interest for reimbursement reasons. We believe this source of error therefore to be negligible. Since the quality reports only contain data from hospitals, the number of surgeries performed in private practice are not included. Due to compensation specifications however, glaucoma surgery in Germany is predominantly performed in hospitals. Still, especially MIGS procedures performed in private practices are a source of bias. Due to the nature of the data source for this study, its scope is limited to broader trends and developments in surgical glaucoma care in Germany. The OPS codes often represent groups of similar procedures and do not allow for in-depth analysis. For example, micro-pulse transscleral laser treatment (mTLT) is not yet listed separately in the OPS system, but is subsumed under OPS code 5–132.22 (transscleral cyclophotocoagulation). For this reason, very new surgical procedures in particular cannot be evaluated in detail. Lastly, the quality reports do not contain data on glaucoma severity or type, which could have influenced surgical decisions during the pandemic.

## Supplementary Information

Below is the link to the electronic supplementary material.

Supplementary file1 (DOCX 21 KB)

## Data Availability

The data analysed during the this study are publicly available from The Federal Joint Committee (G-BA, https://www.g-ba.de/).
