# Supplementary material for: Impact of the COVID-19 pandemic on glaucoma surgery in German hospitals
Source: Graefes Arch Clin Exp Ophthalmol. 2025 Mar 7;263(7):1967–75. doi: 10.1007/s00417-025-06787-9 (PMC12373699; doi:10.1007/s00417-025-06787-9)
Supplement: Supplementary file 1 — Supplementary file1 (DOCX 21 KB) [file 417_2025_6787_MOESM1_ESM.docx]

**Supplementary information**

Full list of all OPS codes that were searched for in the quality reports:

5-131, 5-131.0, 5-131.00, 5-131.01, 5-131.0x, 5-131.1, 5-131.2, 5-131.3, 5-131.4, 5-131.40, 5-131.41, 5-131.42, 5-131.4x, 5-131.5, 5-131.6, 5-131.x, 5-131.y, 5-132, 5-132.0, 5-132.1, 5-132.2, 5-132.x, 5-132.y, 5-133, 5-133.0, 5-133.1, 5-133.2, 5-133.3, 5-133.4, 5-133.5, 5-133.6, 5-133.7, 5-133.x, 5-133.y, 5-134, 5-134.0, 5-134.1, 5-134.x, 5-134.y, 5-133.8, 5-134.2, 5-131.60, 5-131.61, 5-131.6x, 5-131.7, 5-131.8, 5-132.2Zy, 5-132.20, 5-132.21, 5-132.2x, 5-133.9, 5-134.00, 5-134.01, 5-134.10, 5-134.11, 5-133.80, 5-133.81, 5-133.8x, 5-131.62, 5-132.22, 5-131.63, 5-131.64, 5-133.9
